# Genome Sequence of a Novel Picorna-Like RNA Virus from Feces of the Antarctic Fur Seal (*Arctocephalus gazella*)

**DOI:** 10.1128/genomeA.01001-17

**Published:** 2017-09-07

**Authors:** Andi Krumbholz, Marco Groth, Jan Esefeld, Hans-Ulrich Peter, Roland Zell

**Affiliations:** a Institut für Infektionsmedizin der Christian-Albrechts-Universität zu Kiel und des Universitätsklinikums Schleswig-Holstein, Campus Kiel, Kiel, Germany; b CF DNA-Sequenzierung, Leibniz Institut für Alternsforschung, Fritz Lipmann Institut, Jena, Germany; c AG Polar- und Ornitho-Ökologie, Institut für Ökologie, Friedrich-Schiller-Universität Jena, Jena, Germany; d Sektion Experimentelle Virologie, Institut für Medizinische Mikrobiologie, Universitätsklinikum Jena, Friedrich-Schiller-Universität Jena, Jena, Germany

## Abstract

A novel RNA virus was detected in a fecal sample collected from the Antarctic fur seal (*Arctocephalus gazella*) in King George Island, Antarctica. The almost-complete genome sequence reveals two open reading frames and a dicistrovirus-like gene order.

## GENOME ANNOUNCEMENT

Viruses of the *Picornavirales* order are characterized by small nonenveloped capsids and single-stranded RNA genomes with positive-strand polarity encoding one or two polyproteins (1). These are processed by one or more virus-encoded proteinases to yield capsid proteins and nonstructural proteins required for genome replication. Three proteins, a helicase, a chymotrypsin-like proteinase, and an RNA-dependent RNA polymerase—in this order—comprise the so-called replication block and are found in all members of the *Picornavirales*; the gene order is either monocistronic (*Picornaviridae*, *Iflaviridae*, *Marnaviridae*, genus *Waikavirus* of the *Secoviridae*) or dicistronic (*Dicistroviridae*, *Bacillarnavirus*, *Labyrnavirus*) or has bipartite genomes (the *Comovirus*, *Nepovirus*, and *Torradovirus* genera of the *Secoviridae*) (1). Recently, numerous picorna-like viruses with other gene orders were described (2, 3).

Aiming at the investigation of novel viruses of the Antarctic fur seal (*Arctocephalus gazella*) of the South Shetland Islands, Antarctica, fecal samples were collected in the southern summer seasons of 2013/2014 and 2014/2015 near Bellingshausen Station on King George Island. RNA was extracted with the NucliSENS kit (BioMérieux Deutschland GmbH) using an approximately 100-mg stool. Fifty nanograms of RNA extracted from a single sample taken on 15 February 2015 was employed for library preparation using the TruSeq stranded mRNA kit following the manufacturer’s description, except for the following part: in order to address all RNA molecules, purification of poly(A)^+^ molecules was not applied. The library was quality checked and quantified using Agilent’s Bioanalyzer 2100 with the DNA 7500 kit. Sequencing on the Illumina HiSeq2500 platform was done in single-end, 50-cycle mode and yielded 134.92 million reads. Reads were subjected to *de novo* assembly using CLC Workbench software (CLC bio, Aarhus, Denmark). Ninety-one contigs were obtained, six of which exhibited significant similarity to picorna-like viruses in a BLASTx search. Contig sizes ranged from 237 to 846 nucleotides (nt). One contig corresponded to the helicase gene region, two overlapping contigs each to the proteinase gene region and the capsid protein region, respectively, and one contig to the polymerase gene region. Gaps were closed with conventional Sanger sequencing of PCR fragments generated from cDNA with specific oligonucleotides. The genome sequence was extended with 5' and 3' rapid amplification of cDNA ends (RACE).

The genome of the fur seal picorna-like virus comprised 8,116 nt excluding the poly(A) tail. It contains two open reading frames (ORFs) of 4,764 nt and 2,640 nt, respectively, separated by an intergenic sequence of 315 nt. ORF1 is flanked by a 5′ nontranslated region (NTR) of 27 nt and ORF2 by a 3′ NTR of 370 nt plus the poly(A) tail. Preliminary analysis revealed a dicistronic picorna-like virus which is divergent from known dicistroviruses. ORF1 encodes the helicase-proteinase-polymerase proteins, whereas the ORF2 encodes four capsid proteins with similarity to those of dicistroviruses. Conserved sequence elements of the nonstructural protein precursor include a Walker A motif (GxxGxGKS) of the P-loop NTPase superfamily, a GxCx_14_GxHxxG motif of chymotrypsin-like proteinases with an active site cysteine residue, and several motifs of picorna-like RNA-dependent RNA polymerases (KDE, GDYSKYD, PSG, YGDD, and LKR).

### Accession number(s).

The GenBank accession number for the genome of the fur seal picorna-like virus reported here is KY926885.
